# A Case of Therapy-related Acute Myeloid Leukemia Following Treatment with 5-Fluorouracil

**DOI:** 10.7759/cureus.3769

**Published:** 2018-12-24

**Authors:** László Pinczés, Sarolta Molnár, Béla Telek, Árpád Illés

**Affiliations:** 1 Internal Medicine, University of Debrecen, Debrecen, HUN; 2 Pathology, University of Debrecen, Debrecen, HUN

**Keywords:** acute myeloid leukemia, secondary leukemia, 5-fu, antimetabolite, 5-fu

## Abstract

Therapy-related acute myeloid leukemia (t-AML) is most frequently observed after the use of alkylating agents and topoisomerase II inhibitors and is associated with the frequent occurrence of high-risk karyotypes, poor prognosis, and distinct clinical behavior. Therefore, identifying therapy-related causation among patients with newly diagnosed acute leukemia is of great interest. We report the case of a patient who developed therapy-related acute myeloid leukemia after exposure to antimetabolite chemotherapy and emphasize the importance of identifying genetic alterations when the possibility of a therapy-related origin arises.

## Introduction

Therapy-related acute myeloid leukemia (t-AML) and myelodysplastic syndrome (t-MDS) constitute the unique clinicopathologic syndrome of therapy-related myeloid neoplasms (t-MNs). T-MNs form a distinct category within the World Health Organization (WHO) classification, occurring as a late complication after treatment with cytotoxic therapy for a primary neoplastic or non-neoplastic disease. These entities are direct transcriptional consequences of somatic mutations, gained as a result of exposure to antecedent cytotoxic therapy. T-MNs are associated with specific clinical features and a uniformly poor prognosis [1]. T-AML reportedly comprises more than 6% of de novo acute myeloid leukemia (AML) cases, with an increasing incidence due to changes in cancer treatment practices, resulting in increasing numbers of cancer survivors [2].

Therapy-related AML typically occurs after exposure to alkylating agents or topoisomerase II inhibitors. The majority of these cases are seen at least five years after treatment with alkylating agents and are associated with frequent abnormalities of chromosome 5 or 7. Others develop t-AML one to three years after exposure to topoisomerase II inhibitors and often present with translocations involving chromosome 11 or 21 [3]. Although in the past, ionizing radiation was also associated with an elevated risk of t-MNs, the radiotherapy (RT) techniques that have evolved in the last decade cause significantly reduced bone marrow exposure. Nowadays, patients that develop AML after receiving RT alone share genetic features and clinical behavior with de novo AML [4]. This suggests that there is no direct connection between radiation toxicity and leukemogenesis.

To the best of our knowledge, t-AML after exposure to antimetabolites, especially 5-fluorouracil (5-FU), has hardly ever been reported [5-6]. We present the case of a patient diagnosed with AML after neoadjuvant treatment with 5-FU and involved field RT for colorectal carcinoma.

## Case presentation

Our patient was a Caucasian male whose history consisted of laparoscopic cholecystectomy due to cholecystitis acuta calculosa in his early 40s and the diagnosis of hypertension in his late 50s. His family history was free of malignant diseases.

At the age of 66, our patient was diagnosed with stage III colorectal adenocarcinoma (T3N1M0) based on the Union of International Cancer Control (UICC) staging system. He was treated surgically by left hemicolectomy and transverso-rectostomy. The surgical procedure was followed by neoadjuvant chemotherapy with 5-FU monotherapy (5850 mg cumulative dose, CIFU protocol) and the subsequent irradiation of the pelvic area (50.4 Gy cumulative dose, using the conformal technique). He was considered disease-free on consecutive follow-up visits.

Our patient was admitted to the division of hematology at the age of 68, exactly 30 months after treatment for colorectal cancer. He presented with a white blood cell count of 9.8x10^9^/L (normal range: 4.5-10.8x10^9^/L), severe anemia (6.2 g/dL hemoglobin level, normal range: 11.5-15 g/L), and thrombocytopenia (8x10^9^/L thrombocyte count, normal range: 150-400x10^9^/L). The peripheral smear showed 88% blast cells with prominent nucleoli. Liver and kidney function tests were normal. The serum carcinoembryonic antigen (CEA) was within the normal range and no clinical evidence of colorectal cancer recurrence was found. On admission, the patient reported general weakness, dyspnea on exertion, chest pain, and heart palpitations. He had no lymphadenomegaly and no fever or drenching night sweats. He had no abnormal skin lesions and no splenomegaly but mild hepatomegaly was observed.

**Figure 1:**
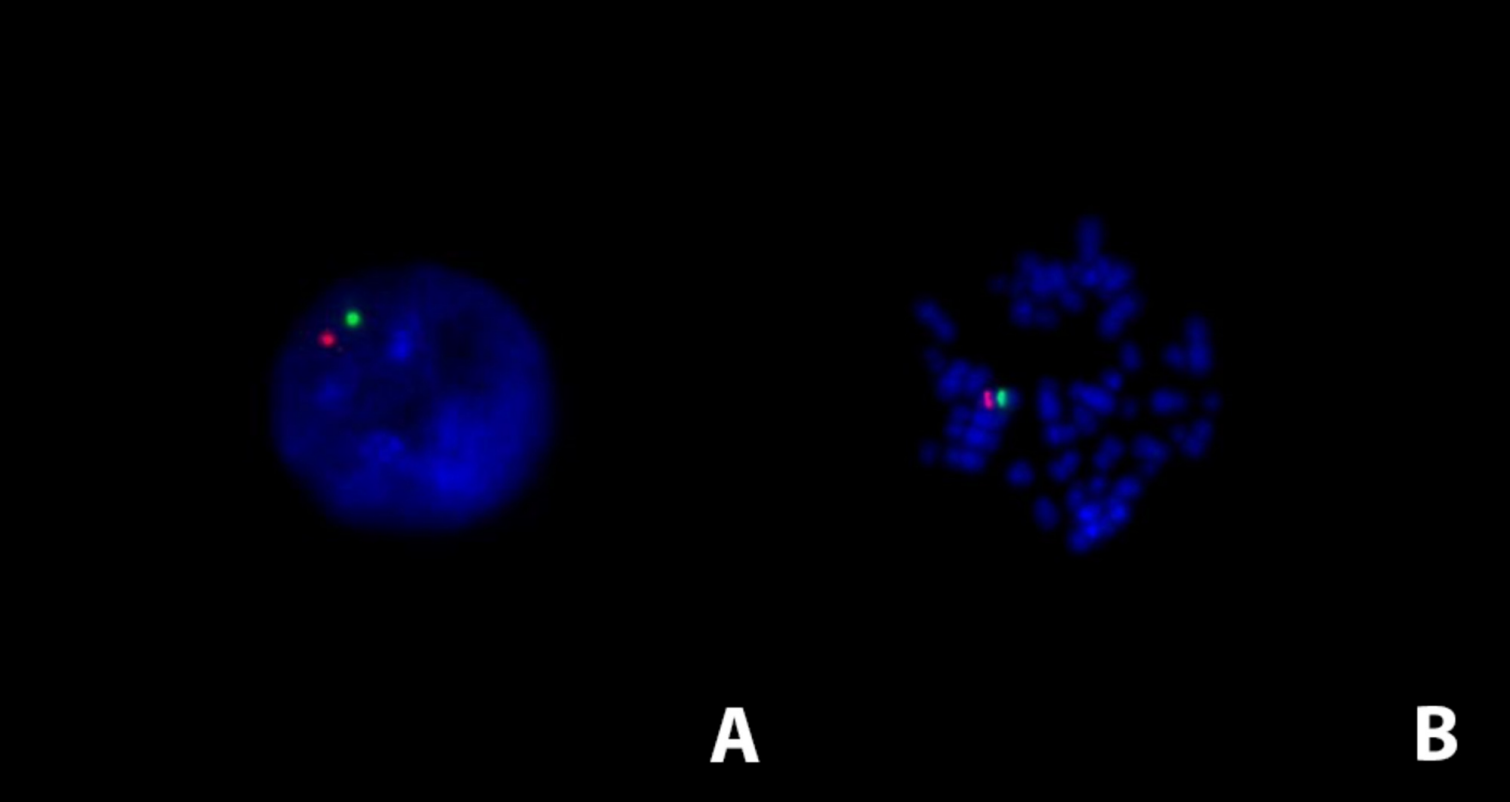
Detection of monosomy 7 by fluorescence in situ hybridization Detection of monosomy 7 using fluorescence in situ hybridization on interphase (A) and metaphase (B) cells. The green signal indicates the centromeric region of chromosome 7, the red signal represents the 7q31 region (LSI CEP7/D7S486, Abbott Molecular). In the case of monosomy 7, only one signal from each probe can be detected.

A bone marrow examination showed a 90% myeloid blast count and depletion of the normal hematopoietic precursors. A diagnosis of AML was made. Results from nucleophosmin-1 (NPM1) and Fms like tyrosine kinase 3 (FLT3) mutational testing identified wild-type alleles but CCAAT/enhancer-binding protein alpha (CEBPA) status were not available. A chromosome banding analysis did not present microscopic clonal alterations. However, the patient’s history of preceding cytotoxic therapy increased the possibility of therapy-related disease. Therefore, fluorescence in situ hybridization (FISH) of bone marrow cells was performed. FISH confirmed clonal monosomy of chromosome 7, an alteration frequently observed in t-AML (Figure 1).

**Figure 2:**
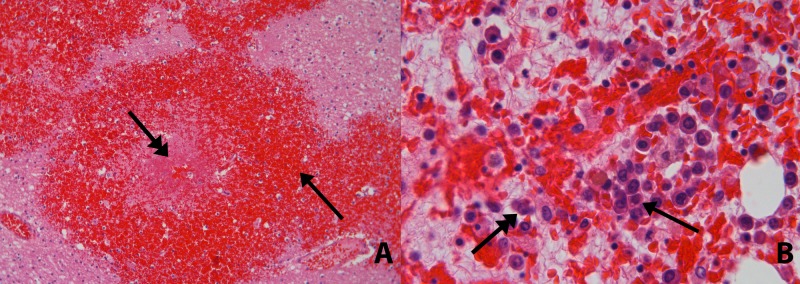
Histological examination Panel A, Brain section (hematoxylin and eosin to 10×) Recent multifocal hemorrhage with areas of necrosis (arrow) and many granulated eosinophil fungal hyphae in the center (double arrow), compatible with Aspergillus. Panel B, Bone marrow (hematoxylin and eosin to 63×). Postmortem sternal bone marrow showing 35% cellularity with myeloid blasts (arrow). Hypocellularity is also contributed to by the praemortal infection. Prominent hemophagocytosis (double arrow) characterized by numerous macrophages, distended with red blood cells, also emphasizes the depletion of myelopoiesis.

Our patient was administered induction chemotherapy under the 3+7 regimen (consisting of 45 mg/m^2^ of daunorubicin and 100 mg/m^2^ of cytarabine). The daunorubicin dose was determined to be 45 mg/m^2^ considering concerns about the tolerability of the higher dose. Subsequent bone marrow testing revealed chemorefractory disease with a nearly 100% blast count. Salvage chemotherapy was administered according to the FLAG-Ida protocol (consisting of 15 mg/m^2^ of fludarabine, 1400 mg/m^2^ of cytarabine, 5 μg/kg filgrastim, and 5 mg/m^2^ idarubicin). Despite the best supportive care, our patient died of septic invasive Aspergillosis, resulting in cerebral Aspergilloma (Figure 2A) and acute bronchopneumonia. Post-mortem bone marrow examination confirmed persisting myeloid blast cells after the salvage protocol (Figure 2B).

## Discussion

Alkylating agents and topoisomerase II inhibitors are well-known factors associated with an increased risk of developing t-AML. However, exposure to any type of mutagenic agents can cause the development of secondary malignancies. Antimetabolites are structural analogs of naturally occurring substances, which are normally incorporated into key molecules, such as deoxyribonucleic acid (DNA) or inhibitors of enzymes involved in DNA synthesis. The concentration required for cytotoxicity decreases with increasing duration of exposure. As an antimetabolite, the pyrimidine analog 5-FU is similar in structure to uracil. This way, it can mimic the natural base and functions after administration to alter the activity of the thymidylate synthetase enzyme, resulting in failure of normal DNA synthesis by inducing p53-dependent cell growth arrest and apoptosis [7]. Selective pressure by cytotoxic therapy on hematopoietic progenitor cells carrying mutations in the TP53 pathway can lead to t-AML [8].

It is important to note that our patient also received radiotherapy during the treatment of his primary neoplastic disease. However, the development of radiation planning capabilities and the use of conformal delivery markedly improved the dose-volume effect on normal tissue, most significantly in the pelvic area [9]. Also, myeloid neoplasms occurring after modern irradiation regimens without the administration of cytotoxic chemotherapy differ from classical t-MNs. The clinical management of these cases is based on de novo disease strategies, which may represent no direct consequence of irradiation in disease development [4]. The RT of our patient was administered using the conformal technique, meaning that radiation-induced DNA damage, leading to t-AML is unlikely.

As in the previously reported t-AML cases after antimetabolite chemotherapy, leukemia arose two to four years after the administration of 5-FU in our setting [5-6]. Furthermore, one patient also presented with a karyotype conveying an adverse prognosis at diagnosis and had refractory disease, not responsive to first-line anthracycline-based therapy. The other reported patient had a cytogenetic abnormality not classified as favorable or adverse and achieved complete remission following induction therapy and was due to receive consolidation therapy at the time. Therefore, the duration of the remission is unknown.

There have been no prospective randomized clinical trials directed specifically at the treatment of t-MNs, thus there is no evidence that any induction therapy would be superior to the standard 3+7 regimen. The management of patients with t-AML is recommended to be guided by cytogenetic and molecular features. However, there is a perception that these patients should be referred to allogeneic transplantation in the first complete remission, due to the high-risk nature of their disease. Of note, the impact of antecedent therapy and subsequent organ damage is impossible to accurately measure. Therefore, increased treatment-related morbidity and mortality may also compromise therapy in t-AML [3]. Also, heavy pre-exposure can lead to the early application of novel treatment modalities, such as hypomethylating agents.

A key factor in our report is the fact that we pursued more precise diagnostic options after receiving negative cytogenetic results in the first place because the clinical possibility of t-AML arose. Confirmation of the high-risk karyotype, according to the European LeukemiaNet (ELN) recommendation, helped us to choose a risk-adapted treatment strategy for our patient [3]. Our patient was not eligible for allogeneic hematopoietic stem cell transplantation at the time, although an adequate candidate in the same clinical setting could be at risk of undertreatment without verification of the genetic aberrations present. We emphasize the gravity of considering the probability of chromosomal rearrangements even at the submicroscopic level, when the possibility of t-AML emerges. Also, treatment failure after induction and salvage chemotherapy emphasizes the aggressive nature of t-AMLs.

It is very difficult to distinguish the cases of AML in which therapy or exposure caused or contributed to developing AML while the development of a second primary malignancy can never be ruled out [10]. However, considering the prior use of a cytotoxic agent, the presentation of one of the most frequently observed genetic alterations in t-AML and the clinical behavior of the therapy refractory disease make the therapy-induced causation likely in our case.

## Conclusions

Therapy-related AML conveys an independent risk factor for poor survival and is associated with a higher frequency of high-risk karyotypes. Therefore, identifying these cases holds huge clinical significance. This report emphasizes the importance of taking the possibility of t-MNs into account in patients with newly diagnosed AML whose medical history includes preceding cytotoxic therapy, regardless of its mechanism of action.
